# [^89^Zr]Oxinate_4_ for long-term in vivo cell tracking by positron emission tomography

**DOI:** 10.1007/s00259-014-2945-x

**Published:** 2014-10-31

**Authors:** Putthiporn Charoenphun, Levente K. Meszaros, Krisanat Chuamsaamarkkee, Ehsan Sharif-Paghaleh, James R. Ballinger, Trevor J. Ferris, Michael J. Went, Gregory E. D. Mullen, Philip J. Blower

**Affiliations:** 1 King’s College London, Division of Imaging Sciences and Biomedical Engineering, 4th Floor Lambeth Wing, St Thomas’ Hospital, London, SE1 7EH UK; 2 School of Physical Sciences, University of Kent, Canterbury, CT2 7NH UK; 3 Division of Chemistry, King’s College London, Britannia House, 7 Trinity St, London, SE11DB UK

**Keywords:** PET, Cell labelling, Cell tracking, ^89^Zr, Leukocyte labelling

## Abstract

**Purpose:**

^111^In (typically as [^111^In]oxinate_3_) is a gold standard radiolabel for cell tracking in humans by scintigraphy. A long half-life positron-emitting radiolabel to serve the same purpose using positron emission tomography (PET) has long been sought. We aimed to develop an ^89^Zr PET tracer for cell labelling and compare it with [^111^In]oxinate_3_ single photon emission computed tomography (SPECT).

**Methods:**

[^89^Zr]Oxinate_4_ was synthesised and its uptake and efflux were measured in vitro in three cell lines and in human leukocytes. The in vivo biodistribution of eGFP-5T33 murine myeloma cells labelled using [^89^Zr]oxinate_4_ or [^111^In]oxinate_3_ was monitored for up to 14 days. ^89^Zr retention by living radiolabelled eGFP-positive cells in vivo was monitored by FACS sorting of liver, spleen and bone marrow cells followed by gamma counting.

**Results:**

Zr labelling was effective in all cell types with yields comparable with ^111^In labelling. Retention of ^89^Zr in cells in vitro after 24 h was significantly better (range 71 to >90 %) than ^111^In (43–52 %). eGFP-5T33 cells in vivo showed the same early biodistribution whether labelled with ^111^In or ^89^Zr (initial pulmonary accumulation followed by migration to liver, spleen and bone marrow), but later translocation of radioactivity to kidneys was much greater for ^111^In. In liver, spleen and bone marrow at least 92 % of ^89^Zr remained associated with eGFP-positive cells after 7 days in vivo.

**Conclusion:**

[^89^Zr]Oxinate_4_ offers a potential solution to the emerging need for a long half-life PET tracer for cell tracking in vivo and deserves further evaluation of its effects on survival and behaviour of different cell types.

**Electronic supplementary material:**

The online version of this article (doi:10.1007/s00259-014-2945-x) contains supplementary material, which is available to authorized users.

## Introduction

Cell tracking by scintigraphy with radionuclides has been routine in nuclear medicine for 30 years [1] for tracking autologous leukocytes to detect sites of infection/inflammation [2, 3]. The standard radiolabelling methodology has been non-specific assimilation of lipophilic, metastable complexes of ^111^In (with oxine [4], tropolone [5] and occasionally other bidentate chelators [6]), and later ^99m^Tc [7].

New insight in immunology is creating interest in imaging the migration of individual immune cell types (e.g. eosinophils [8, 9], neutrophils [8, 9], T-lymphocytes [10–12], and dendritic cells [13]) in cancer, atherosclerosis, stroke, transplant and asthma. Regenerative medicine and cell-based therapies are creating new roles for tracking stem cells and chimeric antigen receptor-expressing T-lymphocytes [14, 15]. Conventional labelling methods have been applied in some of these areas, but for clinical use some of these new applications will require detection of small lesions and small numbers of cells beyond the sensitivity of gamma camera imaging with ^111^In (e.g. coronary artery disease, diabetes, neurovascular inflammation and thrombus), creating a need for positron-emitting radiolabels to exploit the better sensitivity, quantification and resolution of clinical positron emission tomography (PET).

So far the search for positron-emitting radiolabels for cells has met with limited success. The near-ubiquitous presence of glucose transporters allows labelling with [^18^F]fluorodeoxyglucose (FDG), but labelling efficiencies are highly variable, the radiolabel is prone to rapid efflux and the short half-life (110 min) of ^18^F allows only brief tracking [16–18]. ^68^Ga can be used to label cells [19] but it too has a short half-life (68 min). ^64^Cu offers a longer (12 h) half-life and efficient cell labelling using lipophilic tracers (complexes of PTSM [11, 20–22], GTSM [23], diethyldithiocarbamate [24] and tropolonate [25]), but rapid efflux of label from cells is a persistent problem and a still longer half-life would be preferred. A “PET analogue” of [^111^In]oxinate_3_, capable of cell tracking over 7 days or more, would be highly desirable but is not yet available.

^89^Zr is a long half-life positron emitter that could meet this need [26, 27]. The favoured oxidation state of zirconium is 4+ (compared to 3+ for indium), but the parallels between the two metals in reactivity and preferred ligand types suggest that the mechanism exploited to label cells with ^111^In (i.e. lipophilic metastable chelates entering cells and subsequently dissociating) might be exploited in the case of ^89^Zr. Tetravalent zirconium forms ZrL_4_ complexes with monobasic bidentate ligands such as oxinate [28], tropolonate [29] and hydroxamates [30], analogous to InL_3_ [31, 32]. Here we describe the first synthesis of [^89^Zr]oxinate_4_ and comparison with [^111^In]oxinate_3_ for labelling several cell lines and tracking eGFP-5T33 cells in mice. eGFP-5T33 is a syngeneic murine multiple myeloma model originating from the C57Bl/KaLwRij strain [33], engineered to express enhanced green fluorescent protein (eGFP). It was chosen for this work because the fate of the cells after i.v. inoculation is known from the literature [34–36] and prior work in our laboratory. Like human multiple myeloma, 5T33 develops elevated serum immunoglobulin levels and osteolytic disease [33, 37]. Intravenously injected cells migrate exclusively to the liver, spleen and bone marrow [37].

## Materials and methods

### Radiochemistry

^89^Zr was supplied as Zr^4+^ in a 0.1 M oxalic acid (PerkinElmer, Seer Green, UK), brought to pH 7 with 1 M Na_2_CO_3_ and diluted to 500 μl with water. This “neutralised [^89^Zr]oxalate” was used as a control in cell labelling experiments in vitro. To prepare [^89^Zr]oxinate_4_ the above neutralised [^89^Zr]oxalate solution (typically 20–90 MBq) was added to a glass reaction vessel containing 500 μl of a 1 mg/ml 8-hydroxyquinoline solution in chloroform. The vessel was shaken for 15 min and the product [^89^Zr]oxinate_4_ was recovered from the chloroform phase by evaporation, redissolved in dimethyl sulfoxide (DMSO, 10–20 μl) and diluted with phosphate-buffered saline (PBS, 1–3 ml) or cell culture medium. Full details are provided in the Electronic Supplementary Material (ESM).

### Cell labelling initial evaluation

[^89^Zr]Oxinate_4_ was evaluated in vitro in three cell lines: cultured J774 mouse macrophages [38], MDA-MB-231 breast cancer cells [39] and eGFP-5T33 murine myeloma cells [40]; and in leukocytes from healthy volunteers. Studies were approved by an independent UK National Research Ethics Committee and complied with the Declaration of Helsinki. Culture methods and leukocyte preparation and labelling are described in the ESM. Labelling of cell lines was initially evaluated as follows: To triplicate suspensions of 10^6^ cells in 450 μl of serum-depleted [i.e. not supplemented with foetal bovine serum (FBS)] culture medium in glass tubes were added 0.05 MBq of [^89^Zr]oxinate_4_ (or neutralised [^89^Zr]oxalate) in 50 μl of serum-depleted medium. After incubation for up to 60 min at room temperature the tubes were centrifuged (5 min, 490 *g*), 450 μl of supernatant was removed and both supernatant and pellet were counted in a Wallac 1282 Compugamma Universal Gamma Counter. Pellet activity was corrected for the residual 50 μl of supernatant. Similar methods were used with higher activities (up to 40 MBq) and cell numbers (up to 5 x 10^7^) and with [^111^In]oxinate_3_ for comparison. After these initial evaluations a standard labelling incubation period of 30 min was adopted for all subsequent biological evaluation.

### Efflux of radioactivity from cells

Cells were labelled with [^89^Zr]oxinate_4_ or [^111^In]oxinate_3_ in glass tubes (10^6^ cells and 0.05 MBq ^89^Zr or 0.1 MBq ^111^In per tube), washed three times with PBS, resuspended in culture medium (500 μl) and incubated at 37 °C. Samples taken at intervals up to 24 h were centrifuged and pellets and supernatants counted.

### Cell viability

To assess the effect of [^89^Zr]oxinate_4_ on viability of eGFP-5T33 cells, samples of 1.2–1.4 × 10^6^ cells (initially 93 % viable based on trypan blue exclusion) were radiolabelled as described above, washed and incubated in 20 ml of RPMI-1640 (supplemented with 10 % FBS, 200 U/l penicillin, 0.1 g/l streptomycin and 2 mM L-glutamate) in T75 tissue culture flasks at 37 °C (5 % CO_2_). After 24 h, viability of these cells and of controls (treated similarly except for omission of [^89^Zr]oxinate_4_) was determined by trypan blue exclusion.

### Labelling eGFP-5T33 cells for in vivo cell tracking

Cells were labelled with [^111^In]oxinate_3_ or [^89^Zr]oxinate_4_ as described above [4 × 10^6^ and 5 × 10^7^ eGFP-5T33 cells/tube; ca. 0.5 MBq per tube for ex vivo organ counting and up to 40 MBq per tube for PET/single photon emission computed tomography (SPECT) imaging], washed three times with PBS and resuspended in 0.2–1 ml of sterile PBS ready for inoculation. Cell viabilities were determined by trypan blue exclusion. To obtain radiolabelled cell lysates, labelled eGFP-5T33 cells were subjected to three cycles of flash-freezing in liquid nitrogen and rapid heating to 90 °C before resuspending in 200–500 μl PBS and repeatedly passing through 27- and 29-gauge needles until visibly homogeneous.

### Ex vivo cell tracking of eGFP-5T33 murine multiple myeloma

Animal experiments complied with the Animals (Scientific Procedures) Act (UK 1986) and Home Office (UK) guidelines. Male C57Bl/KaLwRij mice, 6–7 weeks old (Harlan, UK) were acclimatised for >7 days with ad libitum access to water and diet. To assess early tissue distribution ten mice were inoculated via the tail vein with 2 × 10^6^ cells labelled with 0.6–0.8 MBq [^89^Zr]oxinate_4_ in 100 μl sterile PBS and culled by cervical dislocation at 9 (*n* = 3), 24 (*n* = 4) and 48 h (*n* = 3) post-injection for ex vivo tissue counting. The longer-term biodistribution of [^89^Zr]oxinate_4_- and [^111^In]oxinate_3_-labelled cells was compared in three mice injected with 0.9 MBq [^89^Zr]oxinate_4_ in 6 × 10^6^ cells and three with 1.7 MBq [^111^In]oxinate_3_ in 5 × 10^6^ cells), culled by cervical dislocation after 7 days. Major thoraco-abdominal organs, the left femur and thigh muscle were excised, weighed and gamma-counted.

### Ex vivo fluorescence-activated cell sorting (FACS)

Mice were culled by CO_2_ asphyxiation 2 (*n* = 3) and 7 days (*n* = 3) after inoculation with 10^7^ eGFP-5T33 cells labelled with 1.5 MBq [^89^Zr]oxinate_4_. Livers, spleens and femora were harvested. Livers and spleens were homogenised. Homogenates were filtered through a 40-μm cell strainer (BD, USA), diluted to 10^7^ cells/ml in ice-cold FACS buffer [1 v/v% FBS, 2 μM ethylenediaminetetraacetate (EDTA) in PBS] and kept on ice. Marrow was flushed from the femora with 5–6 ml of ice-cold FACS buffer, filtered through a 40-μm cell strainer and kept on ice. Samples were analysed on a BD FACSAria III cell sorter (BD, USA) collecting 10,000 each of eGFP-positive and eGFP-negative cells (day 2) and 20,000 each of eGFP-positive and eGFP-negative cells (day 7) for gamma counting.

### PET imaging

Preclinical PET/CT images were acquired in a nanoScan® PET/CT (Mediso, Budapest, Hungary) scanner with mice under isoflurane (2 % in oxygen) anaesthesia, starting 60 s before inoculation with 10^7^ eGFP-5T33 cells labelled with 5 MBq [^89^Zr]oxinate_4_ in a 200-μl bolus via the right lateral tail vein. Scanning was continued for 2 h and repeated 1, 2, 5, 7 and 14 days after inoculation, acquiring a CT scan after each PET scan. Control mice were inoculated with [^89^Zr]oxinate_4_ (4 MBq in 100 μl saline) but no cells, and lysate from 1.1 × 10^6^ cells labelled with 0.9 MBq [^89^Zr]oxinate_4_, and scanned between 5 and 50 min post-injection and at 24 h post-injection.

### SPECT imaging

SPECT scans were acquired for 4 h immediately after inoculation of 10^7^ eGFP-5T33 cells labelled with 10 MBq [^111^In]oxinate_3_ in a 200-μl bolus, using a nanoSPECT/CT (silver upgrade, Mediso, Budapest, Hungary) with four high-resolution multi-pinhole collimators. Further SPECT scans were acquired 1, 2, 3, 5 and 7 days post-injection, each followed by a CT scan. As a control, cell lysate from ca. 1.1 × 10^6^ cells labelled with 1.1 MBq [^111^In]oxinate_3_ was injected intravenously followed by SPECT/CT scanning at 60 and 105 min post-injection.

### Statistics

Statistical significance was tested using a two-tailed Student’s *t* test with significance defined as a confidence level of 95 % or above.

## Results

### Synthesis and quality control of [^89^Zr]oxinate_4_

A biphasic system, with the radiolabel in aqueous solution and oxine in chloroform, was used to synthesise [^89^Zr]oxinate_4_. Yields of radioactivity in the chloroform phase were ca. 60 %. Repeated extraction with further aliquots of oxine solution in chloroform led to increased yields (see ESM). Both chloroform extraction and instant thin-layer chromatography (ITLC, R_f_ =0.9, cf. zero for [^89^Zr]oxalate) before and after dilution of the DMSO solution in saline/serum-depleted RPMI-1640 reproducibly showed radiochemical purity above 99 %.

### Cell labelling

Initial labelling experiments in all cell lines and leukocytes showed rapid accumulation of [^89^Zr]oxinate_4_, approaching plateau levels by 30 min (Fig. S1). In all subsequent labelling experiments a standard labelling incubation time of 30 min was adopted, leading to labelling efficiencies in the range of 40–61 % for up to 9 × 10^7^ cells, as shown in Table 1. By contrast, uptake of neutralised [^89^Zr]oxalate, although increasing with time, was much lower and never exceeded 5 % (Fig. S1).

**Table 1:** Cell labelling efficiencies with [^89^Zr]oxinate_4_ and [^111^In]oxinate_3_ in different cell types

	Cell number (concentration)	Labelling efficiency ^89^Zr	Labelling efficiency ^111^In	Medium
J774 mouse macrophages	10^6^ (2 × 10^6^/ml)	23.1 ± 1.8 (*n* = 3) (not optimised)	–	Cell culture medium (DMEM)
MDA-MB-231 breast cancer	10^6^ (2 × 10^6^/ml)	20.2 ± 3.7 (*n* = 3) (not optimised)	–	Cell culture medium (DMEM)
eGFP-5T33 mouse myeloma	<5 × 10^7^ (<1.3 × 10^7^/ml)	43.2 ± 6.4 (*n* = 3)	96, 82 (*n* = 2)	Cell culture medium (RPMI 1640)
Human leukocytes	9 × 10^7^ (4.5 × 10^7^/ml)	54.3 ± 11.9 (*n* = 3)	46.8 ± 12.3 (*n* = 3)	Saline
	2 × 10^7^ (10^7^/ml)	47.0 ± 10.5 (*n* = 3)	–	Saline

### Efflux from cells

Efflux of ^89^Zr from all three labelled cell lines in vitro was significantly slower than efflux of ^111^In; at 24 h, >90 % of ^89^Zr was retained by J774 macrophages (cf. 51.9 % for ^111^In), >83 % by MDA-MB-231 cells (cf. 43.7 % for ^111^In) and >71 % by eGFP-5T33 cells (Fig. S2). Retention of ^89^Zr in leukocytes after 24 h was 85.1 and 86.9 % (*n* = 2).

### Cell viability

[^89^Zr]Oxinate_4_ labelling reduced viability of eGFP-5T33 cells from 93 % before labelling to 76.3 ± 3.2 % immediately post-labelling [cf. 89.7 ± 1.2 % (*p* = 0.01) for ^89^Zr-free controls], but there was no further significant loss in viability 24 h after labelling (87.3 ± 4.0 %, cf. 89.3 ± 3.0 % for ^89^Zr-free controls, *n* = 3).

### In vivo cell tracking

PET imaging after inoculation of [^89^Zr]oxinate_4_-labelled eGFP-5T33 myeloma cells demonstrated accumulation of cells in lungs at 30 min followed by migration to liver, spleen and bone marrow by 24 h. Ex vivo tissue sampling (Table 2 and Fig. S3) confirmed location of radioactivity predominantly in liver, spleen and bone marrow between 9 and 48 h.

**Table 2:** Tissue distribution of ^89^Zr after i.v. inoculation of [^89^Zr]oxinate_4_-labelled eGFP-5T33 cells in C57Bl/KaLwRij mice

Organ	^89^Zr-oxine	^89^Zr-oxine	^89^Zr-oxine	^89^Zr-oxine	^89^Zr-oxine	^89^Zr-oxine
	9 h (*n* = 3)	9 h (*n* = 3)	24 h (*n* = 4)	24 h (*n* = 4)	48 h (*n* = 3)	48 h (*n* = 3)
	%ID/g	%ID	%ID/g	%ID	%ID/g	%ID
Heart	0.85 ± 0.09	0.18 ± 0.02	0.50 ± 0.13	0.09 ± 0.01	0.32 ± 0.07	0.08 ± 0.02
Lungs	2.28 ± 0.41	0.60 ± 0.11	1.35 ± 0.07	0.41 ± 0.04	0.68 ± 0.06	0.21 ± 0.04
Liver	44.6 ± 10.6	73.9 ± 6.3	42.2 ± 9.1	69.2 ± 7.5	30.9 ± 4.2	57.6 ± 5.3
Spleen	75.0 ± 13.1	5.8 ± 0.80	99.5 ± 11.0	6.6 ± 0.38	54.9 ± 1.2	4.7 ± 0.52
Stomach	0.29 ± 0.08	0.14 ± 0.01	0.45 ± 0.17	0.16 ± 0.08	0.28 ± 0.07	0.12 ± 0.01
Small int.	0.35 ± 0.01	0.34 ± 0.05	0.34 ± 0.02	0.28 ± 0.02	0.27 ± 0.07	0.32 ± 0.04
Large int.	0.34 ± 0.04	0.36 ± 0.04	0.27 ± 0.02	0.29 ± 0.14	0.21 ± 0.04	0.26 ± 0.10
Kidneys	3.9 ± 0.45	1.8 ± 0.15	4.0 ± 0.44	1.65 ± 0.26	3.0 ± 0.43	1.42 ± 0.21
Muscle	0.19 ± 0.03	–	0.21 ± 0.05	–	0.13 ± 0.03	–
Femur	7.1 ± 1.4	0.63 ± 0.08	8.4 ± 0.47	0.76 ± 0.02	5.3 ± 0.85	0.52 ± 0.04
Salivary gl.	0.54 ± 0.06	0.12 ± 0.004	0.52 ± 0.14	0.10 ± 0.02	0.44 ± 0.04	0.10 ± 0.03
Blood	1.8 ± 0.18	–	1.0 ± 0.18	–	0.53 ± 0.18	–
Liver:spleen	0.60 ± 0.18	12.7 ± 2.1	0.42 ± 0.10	10.5 ± 1.3	0.56 ± 0.08	12.1 ± 1.7
Liver:femur	6.28 ± 1.94	116.8 ± 17.2	5.02 ± 1.12	91.1 ± 10.2	5.83 ± 1.23	110.8 ± 13.5
Spleen:femur	10.6 ± 2.78	9.2 ± 1.7	11.9 ± 1.47	8.7 ± 0.56	10.4 ± 1.68	9.1 ± 1.22
Liver:kidneys	11.4 ± 3.0	40.9 ± 4.8	10.6 ± 2.6	42.0 ± 8.1	10.3 ± 2.0	40.5 ± 7.1
Spleen:kidneys	19.2 ± 4.0	3.2 ± 0.52	24.9 ± 3.9	4.0 ± 0.67	18.3 ± 2.7	3.3 ± 0.61
Femur:kidneys	1.8 ± 0.42	0.35 ± 0.05	2.1 ± 0.26	0.46 ± 0.07	1.8 ± 0.38	0.37 ± 0.06

%ID and %ID/g were calculated after ex vivo tissue counting; mean ± SD

**Fig. 1:**
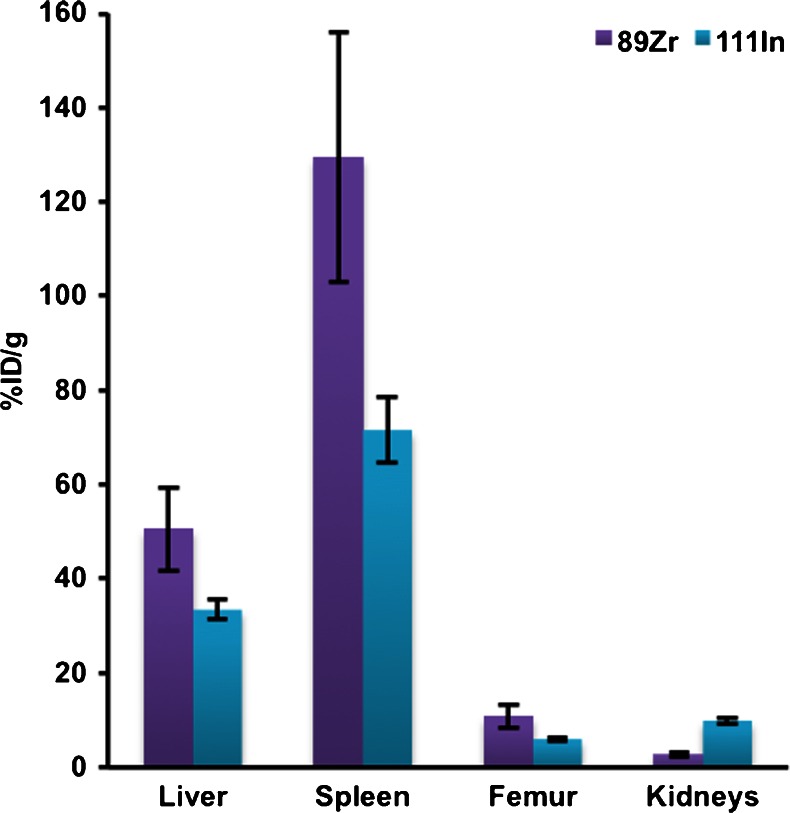
Radioactivity distribution 7 days after inoculation of mice with [^89^Zr]oxinate_4_- and [^111^In]oxinate_3_-labelled eGFP-5T33 cells. %ID and %ID/g were calculated after ex vivo tissue counting and are given as mean ± SD (*n* = 3 per group). Data are selected from Table 3 to show accumulation of ^89^Zr and ^111^In in haemopoietic tissues and kidneys (the main locations of radioactivity)

**Fig. 2:**
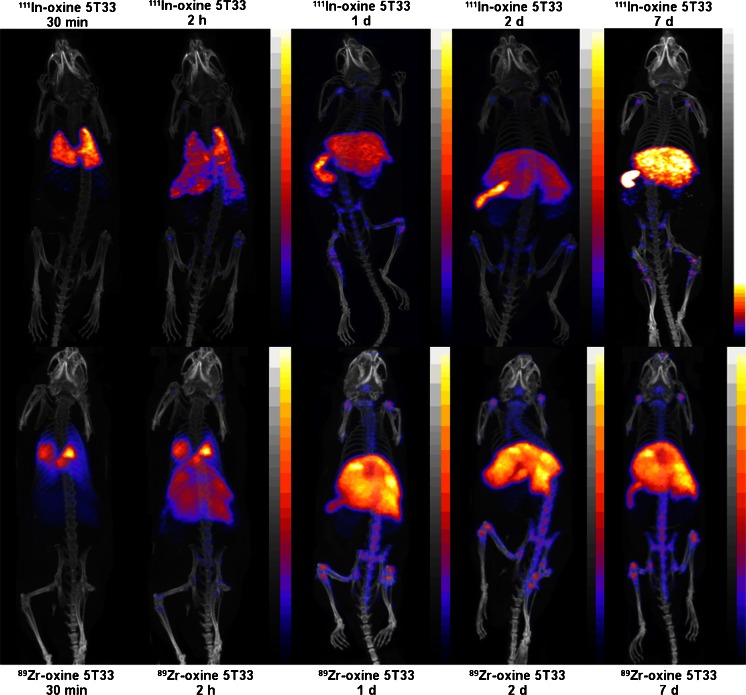
PET/CT and SPECT/CT images of C57Bl/KaLwRij mice inoculated with [^89^Zr]oxinate_4_- or [^111^In]oxinate_3_-labelled eGFP-5T33 cells. Scans of ^111^In (*top*) and ^89^Zr (*bottom*) are reported from 30 min to 7 days after i.v. inoculation of C57Bl/KaLwRij mice with 10^7^ eGFP-5T33 cells labelled with 5 MBq [^89^Zr]oxinate_4_ or 10 MBq [^111^In]oxinate_3_. Image scales were adjusted to the maximum activity concentration within each image except for the 2-h images where the activity scale was adjusted to the maximum activity per voxel used in the 30-min image in order to quantitatively visualise the rapid change in the early homing pattern of eGFP-5T33 cells after i.v. inoculation

The in vivo biodistribution of ^89^Zr-labelled eGFP-5T33 myeloma cells was compared alongside that of cells from the same culture labelled with ^111^In longitudinally over 7 days using PET and SPECT imaging and ex vivo tissue sampling up to 7 days (Fig. 1, Table 3). Cell viabilities were 85–95 % with both radiolabels before inoculation. PET and SPECT images between 30 min and 7 days are shown in Fig. 2. A PET image acquired on day 14 is shown in Fig. S4 and ex vivo tissue uptake data for both radionuclides at 7 days are listed in Table 3. ^111^In SPECT and ^89^Zr PET showed qualitatively similar migration patterns in the first few hours after inoculation. However, there was significantly higher %ID/g of ^89^Zr than ^111^In in the main target organs (liver, spleen, bone) at 7 days (by factors of 1.5, 1.8 and 1.8, *p* = 0.03, *p* = 0.02 and *p* = 0.03, respectively) (Table 3). The %ID/g of ^111^In in kidneys increased with time, reaching 3.75-fold higher (*p* < 0.0001) than that of ^89^Zr by 7 days.

**Table 3:** Radioactivity distribution 7 days after i.v. inoculation of C57Bl/KaLwRij mice with [^89^Zr]oxinate_4_- and [^111^In]oxinate_3_-labelled eGFP-5T33 cells

Organ	^89^Zr-oxine	^89^Zr-oxine	^111^In-oxine	^111^In-oxine
	7 days (*n* = 3)	7 days (*n* = 3)	7 days (*n* = 3)	7 days (*n* = 3)
	%ID/g	%ID	%ID/g	%ID
Heart	0.24 ± 0.04	0.03 ± 0.005	0.71 ± 0.12	0.12 ± 0.02
Lungs	0.35 ± 0.04	0.09 ± 0.009	0.66 ± 0.05	0.20 ± 0.009
Liver	50.6 ± 8.9	58.2 ± 5.5	33.4 ± 2.1	43.8 ± 3.1
Spleen	129.5 ± 26.5	7.9 ± 0.76	71.6 ± 6.9	4.59 ± 0.34
Stomach	0.43 ± 0.12	0.12 ± 0.06	0.66 ± 0.14	0.19 ± 0.03
Small int.	0.17 ± 0.07	0.16 ± 0.06	0.43 ± 0.11	0.44 ± 0.12
Large int.	0.17 ± 0.08	0.18 ± 0.09	0.45 ± 0.07	0.58 ± 0.16
Kidneys	2.64 ± 0.39	0.96 ± 0.06	9.9 ± 0.64	4.2 ± 0.39
Muscle	0.16 ± 0.02	–	0.43 ± 0.05	–
Femur	10.8 ± 2.5	0.88 ± 0.12	6.0 ± 0.5	0.56 ± 0.04
Salivary gl.	0.40 ± 0.03	0.06 ± 0.01	1.96 ± 0.24	0.35 ± 0.08
Blood	0.23 ± 0.1	–	0.22 ± 0.06	–
Liver:spleen	0.39 ± 0.11	7.3 ± 1.0	0.47 ± 0.05	9.5 ± 1.0
Liver:femur	4.69 ± 1.36	66.0 ± 11.2	5.57 ± 0.58	78.7 ± 8.4
Spleen:femur	12.0 ± 3.71	9.0 ± 1.5	11.9 ± 1.52	8.3 ± 0.9
Liver:kidneys	19.2 ± 4.4	60.6 ± 6.7	3.4 ± 0.4	10.5 ± 1.25
Spleen:kidneys	49.1 ± 12.4	8.3 ± 0.92	7.23 ± 0.8	1.1 ± 0.13
Femur:kidneys	4.1 ± 1.1	0.92 ± 0.14	0.61 ± 0.06	0.13 ± 0.02

%ID and %ID/g were calculated after ex vivo tissue counting and are given as mean ± SD (*n* = 3 per group)

**Fig. 3:**
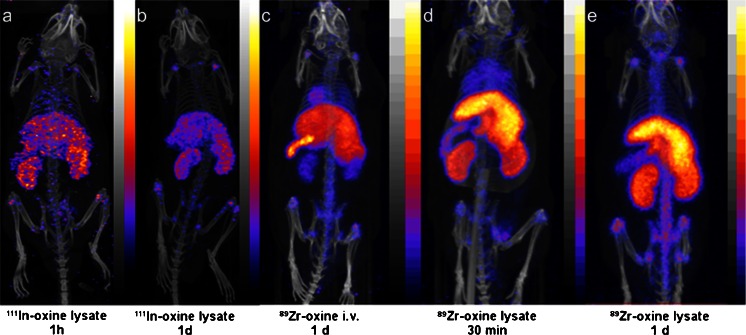
SPECT/CT and PET/CT images of mice injected with radiolabelled eGFP-5T33 cell lysates or [^89^Zr]oxinate_4_. **a**, **b** SPECT/CT images of mice injected with lysates of 1.1 × 10^6^ eGFP-5T33 cells labelled with 1.1 MBq [^111^In]oxinate_3_ at 1 and 24 h post-injection, respectively. **c** PET/CT image of mouse 24 h after injection with 4 MBq [^89^Zr]oxinate_4_. **d**, **e** PET/CT images of mice injected with lysates of 1.1 × 10^6^ eGFP-5T33 cells labelled with 0.9 MBq [^89^Zr]oxinate_4_ at 1 and 24 h post-injection, respectively

When radiolabelled cell lysates were injected i.v., uptake in the liver, spleen and skeleton was observed but there was also a much more marked accumulation in the kidneys, both for ^111^In and ^89^Zr (Fig. 3). Intravenously injected [^89^Zr]oxinate_4_ gave a qualitatively similar biodistribution to labelled cell lysates but there was also significant uptake in the heart and lungs after 24 h.

**Fig. 4:**
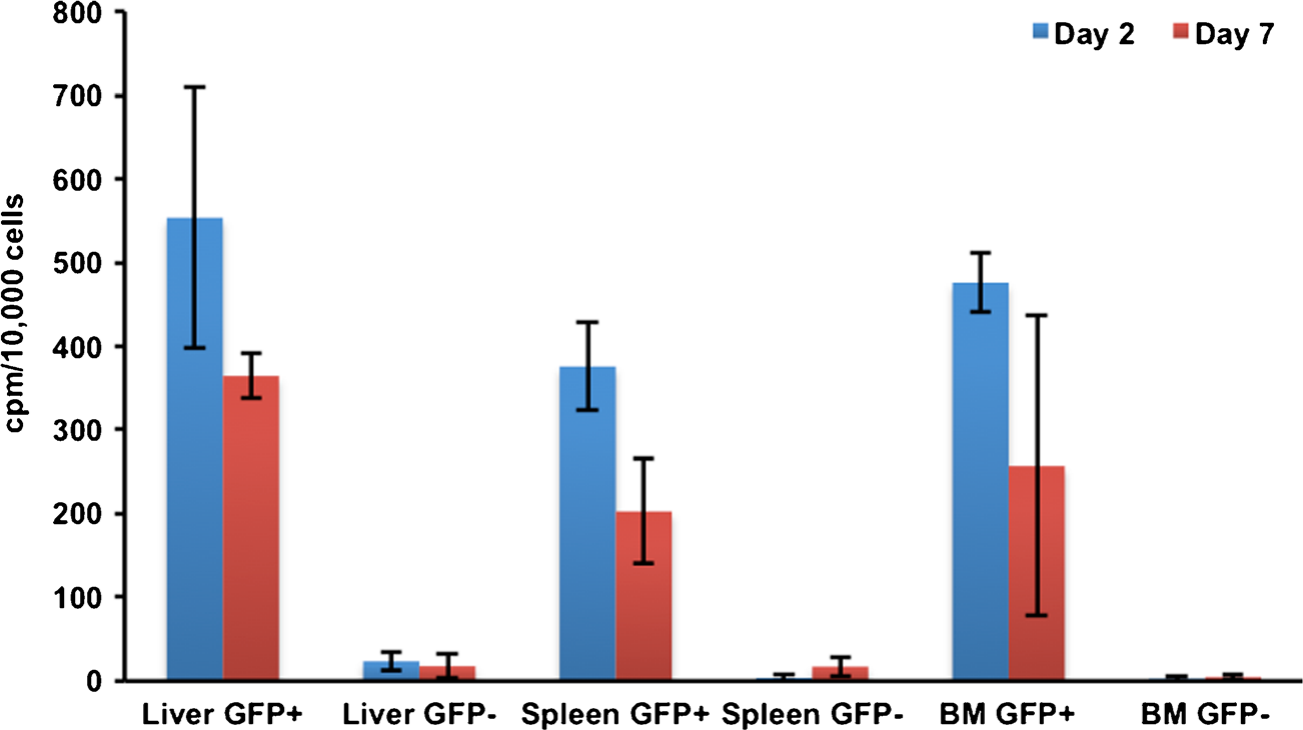
^89^Zr activities in eGFP-positive and eGFP-negative cell populations sorted from organ samples. Liver, spleen and femoral marrow (*BM*) organ homogenates were harvested from mice 2 and 7 days after i.v. inoculation with [^89^Zr]oxinate_4_-labelled eGFP-5T33 cells (*n* = 3/group, decay corrected)

FACS sorting (based on eGFP fluorescence) of organ homogenates from animals culled 2 and 7 days after inoculation with cells labelled with [^89^Zr]oxinate_4_ showed that the eGFP-positive cells contained more activity per cell (by a factor of >23 at 2 days and a factor of >12 at 7 days) than eGFP-negative cells (*p* < 0.02) (Fig. 4). Example FACS data are reported in Fig. S5.

## Discussion

A general-purpose radiolabel that allows the advantages of PET to be implemented for tracking cells in vivo over several days should satisfy several criteria: the radioisotope must have a suitably long half-life and appropriate positron energy and abundance, convenient and economic availability, a simple and efficient labelling procedure, no major selectivity for different cell types, minimal effects on cell survival and function in vivo and minimal efflux from cells. These criteria should be met at least as well as [^111^In]oxinate_3_ meets them and better than positron-emitting alternatives reported to date. We have synthesised a lipophilic, metastable complex, [^89^Zr]oxinate_4_, evaluated it biologically in a range of cell lines in vitro and selected one cell line (eGFP-5T33 myeloma cells) for evaluation in vivo. The results indicate that [^89^Zr]oxinate_4_ meets these criteria. The half-life of ^89^Zr is 3.3 days (longer than that of ^111^In and potential positron-emitting competitors ^64^Cu, ^18^F and ^68^Ga); only ^124^I (4.2 days) is comparable in this respect but cell labelling with ^124^I has not been reported. The positron abundance and energies and gamma emissions of ^89^Zr, although not ideal compared to ^18^F, ^64^Cu and ^68^Ga, proved adequate to give better-resolved images than SPECT with ^99m^Tc in a direct comparison (human sentinel node imaging) [41]. ^89^Zr is now commercially and economically available as a GMP product. The cell labelling procedure described here is simple, indeed identical to that used with [^111^In]oxinate_3_; good labelling efficiencies are achieved within a few minutes. The labelling method is effective for several different cell lines and human leukocytes. The survival of labelled eGFP-5T33 cells in vitro is comparable to or better than that of [^111^In]oxinate_3_-labelled cells and not significantly different to that of control cells treated identically except for omission of radioactivity. While leukocytes are efficiently labelled and show good retention of radioactivity at 24 h, the effects of labelling on survival and function have not been determined, and further investigation of these aspects in the different leukocyte types are required. The in vivo biodistribution and FACS sorting data suggest that labelled eGFP-5T33 cells remain viable for at least 7 days, since the ^89^Zr-labelled cells continue to express eGFP after this period in vivo. Retention of ^89^Zr by cells studied here in vitro over 24 h compares well with published cell radiolabels; it is significantly better than that of ^111^In and markedly superior to that of cells labelled with ^64^Cu complexes of PTSM [11, 20–22] and GTSM [23] or with ^18^F-FDG. The superior retention of ^89^Zr compared to ^111^In by eGFP-5T33 cells in vivo is inferred from the much slower transfer of ^89^Zr than ^111^In from liver, spleen and bone marrow to kidneys and suggests that replacing ^111^In SPECT with ^89^Zr PET can extend the period over which cells can be reliably tracked in vivo; indeed, we have been able to acquire good PET images up to 2 weeks post-inoculation. The 14-day image showed a similar biodistribution of radioactivity to that seen at 7 days in the same mouse suggesting that the radiolabel continued to be retained by 5T33 cells in vivo (Fig. S4).

The overall biodistribution observed with both ^111^In- and ^89^Zr-labelled cells (initial uptake in lung followed by migration to liver, spleen and bone marrow) is consistent with earlier studies of the migration of related murine myeloma cells, by histology and by radiolabelling with ^51^Cr [35]. Organ to organ uptake ratios among the main target organs (liver, spleen, bone marrow) did not vary significantly throughout the study, either for ^89^Zr or ^111^In, suggesting that labelled cells, or radioactivity, did not migrate from one haematopoietic tissue to another and that the radiolabel used did not change the tissue preference of eGFP-5T33 cells. To test the implicit assumption that migration of radioactivity from liver, spleen and bone marrow to kidney and bladder is an indicator of gradual efflux of radioactivity from living or dead labelled cells, we examined the distribution of activity after injection of [^89^Zr]oxinate_4_ and that of cells that had been killed/lysed after radiolabelling with ^89^Zr and ^111^In. The resulting biodistribution qualitatively resembled that seen with labelled healthy cells, but both ^89^Zr- and ^111^In-labelled cell lysate showed greatly increased uptake in kidney and also liver and reduced uptake in spleen, even at early time points (30 min, 24 h, Fig. 3), compared to labelled healthy cells. This contrast in behaviour of ^89^Zr injected in the form of living labelled cells and dead/lysed labelled cells is consistent with the hypothesis that migration of radioactivity from initially targeted organs to kidney and bladder is a non-invasive marker for efflux of radioactivity from living or dying labelled cells in vivo.

^89^Zr injected in the form of neutralised [^89^Zr]-oxalate also shows marked differences from the behaviour of labelled cells, consistent with the assumption that the images obtained after injection of ^89^Zr-labelled cells reflect the location of the cells. With [^89^Zr]oxinate_4_ significant uptake in heart was observed that was not seen with labelled cells, while [^89^Zr]oxalate shows gradual skeletal accumulation: radioactivity can be imaged in the joints as early as 15 min post-injection [42, 43].

The novel experimental approach of ex vivo FACS sorting of eGFP-positive and eGFP-negative populations from organ homogenates, followed by radiation counting of these fractions, showed that the radioactivity in the target tissues remains associated with the originally labelled eGFP-expressing cells (Fig. 4), and hence that these labelled cells remain alive during the 7 days in vivo; 96 % of liver radioactivity, 99 % of spleen radioactivity and >99 % of bone marrow radioactivity remains associated with eGFP-positive cells at 2 days, falling to 95, 92 and 98 %, respectively, by 7 days. Although we observed excellent in vivo survival of labelled eGFP-5T33 cells in this study, a more thorough understanding of the possible cytotoxic effects of the wider use of both ^89^Zr and ^111^In labelling of cells is required for a proper interpretation of cell tracking data generally. This ex vivo FACS-based method provides a potential experimental approach to this challenging problem.

These highly promising results warrant further development including refinement of the radiosynthesis and labelling process to improve radiochemical and cell labelling yields, select the most suitable medium for cell labelling and eliminate undesirable use of chloroform, and determine the functional and survival effects of labelling in a variety of cell types, prior to evaluation of the method for cell tracking in humans. While estimates of likely dosimetry in humans have not yet been performed, it will also be important to assess the potential advantages of cell tracking by PET with ^89^Zr against the likely increased radiation burden per megabecquerel associated with ^89^Zr, balancing the increased dose per decay, the low positron yield of ^89^Zr and the increased detection efficiency of PET compared to SPECT.

## Conclusion

The new lipophilic, metastable complex of ^89^Zr can radiolabel a range of cells, independently of specific phenotypes, providing a long-sought solution to the unmet need for a long half-life positron-emitting radiolabel to replace ^111^In for cell migration imaging. In addition to the expected advantages (enhanced sensitivity, resolution and quantification) of cell tracking with PET rather than scintigraphy or SPECT, ^89^Zr shows less efflux from eGFP-5T33 cells in vitro and in vivo than ^111^In during at least 7 days after labelling. The excellent in vivo survival and retention of radioactivity by the cells at 7 days, coupled with the demonstrated ability to acquire useful PET images up to 14 days, significantly extend the typical period over which cells can be tracked by radionuclide imaging with directly labelled cells. This method offers a potential solution to the emerging need for a long half-life PET tracer for cell tracking in vivo and deserves further evaluation of its effects on survival and behaviour of different cell types, and the dosimetric and radiobiological implications of application in humans.

## Electronic supplementary material

Below is the link to the electronic supplementary material.

ESM 1 (PDF 649 kb)
