# Supplementary material for: [89Zr]Oxinate4 for long-term in vivo cell tracking by positron emission tomography
Source: Eur J Nucl Med Mol Imaging. 2014 Oct 31;42(2):278–87. doi: 10.1007/s00259-014-2945-x (PMC4315484; doi:10.1007/s00259-014-2945-x)
Supplement: Supplementary file 1 — (PDF 649 kb) [file 259_2014_2945_MOESM1_ESM.pdf]

# [$^{89}$Zr]-Zr(oxinate)$_4$ for long term *in vivo* cell tracking by positron emission tomography

(Submitted to the European Journal of Nuclear Medicine and Molecular Imaging)

Putthiporn Charoenphun[†1], Levente K Meszaros[†1], Krisanat Chuamsaamarkkee[1], Ehsan Sharif-Paghaleh[1], James R Ballinger[1], Trevor J Ferris[2], Michael J Went[2], Gregory ED Mullen[1], Philip J Blower*[1,3]

[1]King's College London, Division of Imaging Sciences and Biomedical Engineering, 4th Floor Lambeth Wing, St Thomas' Hospital, London SE1 7EH, UK.

[2]University of Kent, School of Physical Sciences, Canterbury CT2 7NH, UK

[3]King's College London, Division of Chemistry, Britannia House, 7 Trinity St, London SE11DB, UK

[†]PC and LKM contributed equally to this work and are joint first authors.

*Author for correspondence: Prof Phil Blower, King's College London, Division of Imaging Sciences and Biomedical Engineering, 4th Floor Lambeth Wing, St Thomas' Hospital, London SE1 7EH, UK. Tel: +44-207-188-9513; fax: +44-207-188-5442; email: Philip.Blower@kcl.ac.uk

**Synthesis of [$^{89}$Zr]-Zr(oxinate)$_4$**

$^{89}$Zr was supplied as $Zr^{4+}$ in a 0.1M oxalic acid (Perkin-Elmer, Seer Green, UK), brought to pH 7 with 1M $Na_2CO_3$ and diluted to 500 µl with distilled water. This "neutralised [$^{89}$Zr]-Zr-oxalate" was used both as a control in cell labelling experiments *in vitro*, and to prepare [$^{89}$Zr]-Zr(oxinate)$_4$ as follows: 8-hydroxyquinoline (oxine) (Sigma, Gillingham, UK) in chloroform (500 µl, 1 mg/ml) was added to a glass reaction vessel containing 500 µl (20-90 MBq, measured with a Capintec CRC-25 dose calibrator) of neutralised [$^{89}$Zr]-Zr-oxalate The vessel was shaken (vortexed, 1000 RPM) for 15 minutes to facilitate phase transfer. The two phases were then allowed to separate and the aqueous phase was transferred into a separate vessel. The chloroform extract was evaporated at 60 °C and the residue containing [$^{89}$Zr]-Zr(oxinate)$_4$ was dissolved in 10-20 µl of DMSO and diluted to 1-3 ml with PBS or cell culture medium. In some experiments the used aqueous phase was extracted again with further aliquots of oxine solution in chloroform to increase the yield. Quality control was performed with instant thin layer chromatography (ITLC) and solvent extraction as described below.

**Quality control of [$^{89}$Zr]-Zr(oxinate)$_4$**

ITLC: Samples were analysed on silica gel coated ITLC strips (ITLC-SG; Agilent, UK) with ethyl acetate mobile phase. 8-9 cm long strips were developed in 50 ml centrifuge tubes. Chromatograms were evaluated on a LabLogic MINI-SCAN radio TLC linear scanner connected to a LabLogic B-FC-3200 NaI detector for gamma photon detection.

Solvent extraction: A biphasic solvent system of phosphate buffered saline (PBS) and chloroform (1:1) was prepared 24 hours before the study to allow equilibration (PBS saturated in chloroform and *vice versa*). Five hundred µl of both phases were transferred into a 1.5 ml microcentrifuge tube. To this mixture 0.5 µl of [$^{89}$Zr]-Zr(oxinate)$_4$ in DMSO or 0.5 µl of neutralised $^{89}$Zr in water were added. The mixtures were vortexed at a high speed for 1

minute. Upon separation of the two phases 100 μl of each phase were transferred into a scintillation vial and activities associated with the organic and aqueous phase were determined in the gamma counter.

**Cell culture**

The J774 cell line was kindly provided by Dr. Helen Collins, Department of Infectious Diseases, King's College London. Cells were cultured in Dulbecco's Modified Eagle's Medium (DMEM, 4.5 g/l glucose) supplemented with 10% foetal bovine serum (FBS), 2mM L-glutamine, penicillin (100 U/ml), streptomycin (0.1 mg/ml), 1 mM sodium pyruvate and 10 mM HEPES. MDA-MB-231 cells were cultured in DMEM (4.5 g/l glucose) supplemented with 10% foetal bovine serum, 2mM L-glutamine, penicillin (100 U/ml) and streptomycin (0.1 mg/ml). Cell lines were cultured in a tissue culture incubator with humidified 95% air 5% $CO_2$ atmosphere at a constant temperature of 37°C. Both cell lines were grown in adherent cultures in T75 or T175 cell culture flasks and split twice a week (typically when full confluence was reached). Cells were harvested from culture as follows. Supernatant was discarded and cells were washed twice with PBS. To obtain J774 cells culture medium (complete medium for culture maintenance and FBS depleted for labelling) was added and cells were removed from the plastic surface by gentle scraping. MDA-MB-231 cells were trypsinised as follows. 1-2 ml of trypsin-EDTA (containing 0.5 g/l porcine trypsin and 0.2 g/l EDTA in Hank's Balanced Salt Solution) were added to the cell culture flask and dispersed on the plastic surface. The flask was then incubated in a tissue culture incubator for 5 min. Cells were obtained by adding at least 20 ml of medium to the flask then diluted to the required concentration.

The eGFP-5T33 murine multiple myeloma cell line was provided by Dr Yolanda Calle (University of Roehampton). This cell line was used for *in vivo* cell tracking. Cells were grown in suspension culture in RPMI 1640 supplemented with 10% FBS, 2 mM L-glutamine,

penicillin (100 U/ml) and streptomycin (0.1 mg/ml). This cell line was maintained in suspension culture.

When cells were prepared for labelling with one of the radiotracers they were repeatedly washed by resuspending in PBS and centrifuging (5 min, 490 g; three times in total) to remove residues of serum and other culture medium constituents.

**White blood cell separation and radiolabelling**

These studies were approved by an independent United Kingdom national research ethics committee. This procedure (approximating a standard method used clinically for $^{111}$In-labelling - not identical to that provided in EANM guidelines[1]), was carried out on three different occasions. Fifty ml blood was withdrawn from a peripheral blood of a healthy volunteer as follows: 500 U heparin (0.5 ml) were withdrawn into a 50 ml syringe. The syringe was then fitted with a 20 gauge scalp vein set and 50 ml blood was slowly withdrawn from a peripheral vein of the volunteer. Upon completion the scalp vein set was replaced with a luer lock syringe cap to seal the syringe. The syringe was gently turned around several times to mix its contents. The syringe was then transferred into a class 2 tissue culture cabinet where WBC separation was performed aseptically as follows. Two ml of Methocel 2% and 40 ml of whole blood were added to a 50 ml centrifuge tube. The tube was sealed then contents were mixed gently. The remaining 10 ml of whole blood were transferred to a 15 ml centrifuge tube. Both tubes were placed in a plastic rack in upright position. To separate white blood cells for subsequent radiolabelling the 50 ml centrifuge tube was centrifuged at 4.9 g for 15 min. After centrifugation, the supernatant containing white blood cells, platelets and leukocyte rich plasma (LRP) was transferred into a new centrifuge tube and centrifuged at 91 g for 5 min. After centrifugation, white blood cells formed a pellet at the bottom of the tube, the supernatant contained platelet-rich plasma (PRP). The supernatant was discarded and the pellet was re-suspended in 10 ml of isotonic saline. The tube was centrifuged at 91 g

for 5 min then the supernatant was discarded. The pellet was re-suspended in 1 ml of saline then transferred into a glass test tube. [$^{89}$Zr]-Zr(oxinate)$_4$ in 15 µl DMSO was diluted in 1 ml normal saline then added to the above cell suspension and mixed very gently. Cells were incubated with the tracer at room temperature for 15 min. The experiment was repeated three times with different levels of activity (3.05, 11.10, and 16.10 MBq). In the meantime platelet poor plasma (PPP) was prepared from 10 ml whole blood by centrifuging the sample at 490 g for 5 min. Supernatant layer containing platelet-poor plasma was collected in a fresh centrifuge tube. PPP was later used to dilute and re-suspend WBCs after radiolabelling. After incubating WBCs with the radiotracer the labelled WBC suspension was transferred into a 50 ml centrifuge tube. Three ml of PPP were added to the WBC suspension which was then diluted to 10 ml in saline. The resulting suspension was centrifuged at 91 g for 5 min. The supernatant containing unbound [$^{89}$Zr]-Zr(oxinate)$_4$ was transferred into a fresh tube. The radiolabelled WBC pellet was gently re-suspended in 3 ml of PPP. Activity of the labelled cells and supernatant (unreacted tracer) were measured in the dose calibrator. The labelled cell suspension in PPP was transferred into a glass test tube and incubated in a tissue culture incubator for 24 h when retention of the radiotracer was assessed after centrifugation and separation of the supernatant and labelled WBC pellet.

References

1. Roca M, de Vries EFJ, Jamar F, Israel O, Signore A. Guidelines for the labelling of leucocytes with 111In-oxine. *Eur J Nucl Med Mol Imaging*. 2010; 37: 835–841.

**Figures**

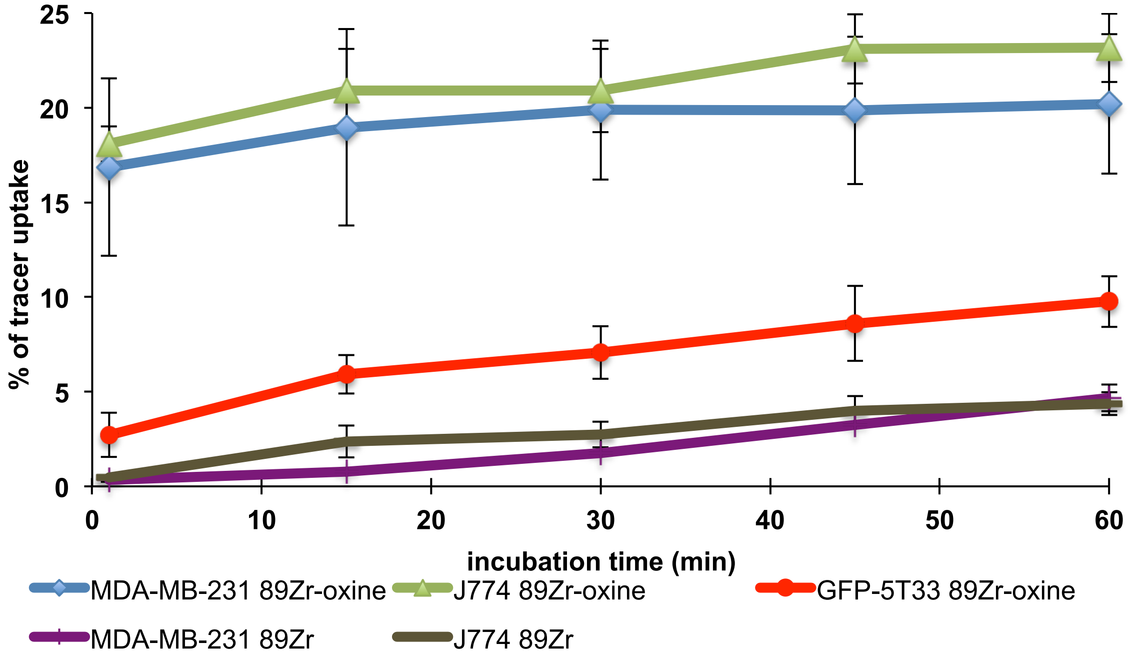


**Fig. S1** Results of preliminary labelling experiments to investigate time course of uptake in cells. Cellular uptake in three cell lines of [$^{89}$Zr]-Zr(oxinate)$_4$ ("$^{89}$Zr-oxine") and neutralised [$^{89}$Zr]-Zr-oxalate ("$^{89}$Zr") ($10^6$ cells were incubated with 0.05 MBq of each tracer, in triplicate; mean ± SD)

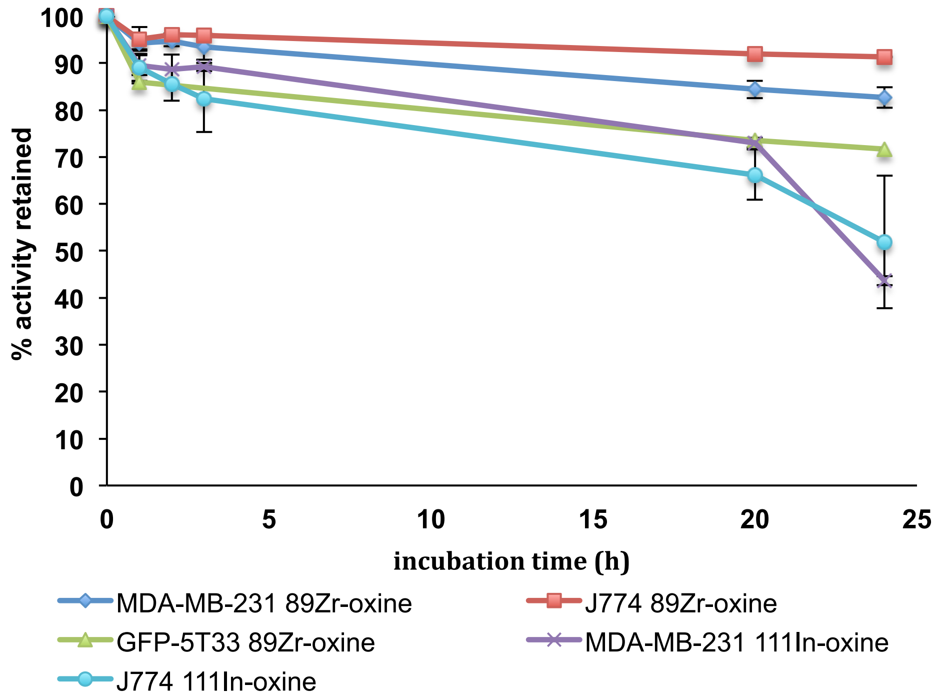


**Fig. S2** Cellular retention *in vitro* of [$^{89}$Zr]-Zr(oxinate)$_4$ ("$^{89}$Zr-oxine") and [$^{111}$In]-In(oxinate)$_3$ ("$^{111}$In-oxine") (n=3/time point, mean ± SD, 100% refers to activity in washed cells at the end of the labelling process)

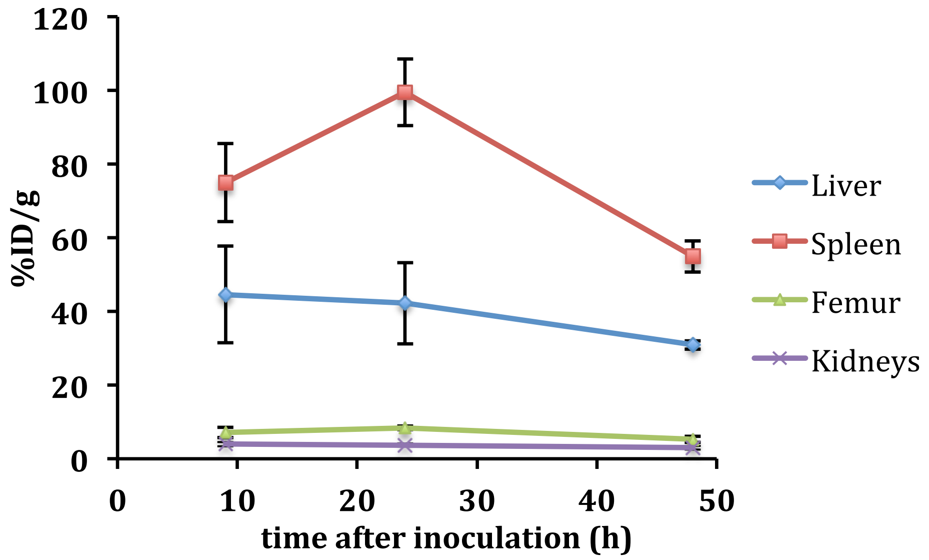


**Fig. S3** Accumulation of [$^{89}$Zr]-Zr(oxinate)$_4$ labelled eGFP-5T33 cells in major sites of uptake in C57Bl/KaLwRij mice after i.v. inoculation (mean ± SD)

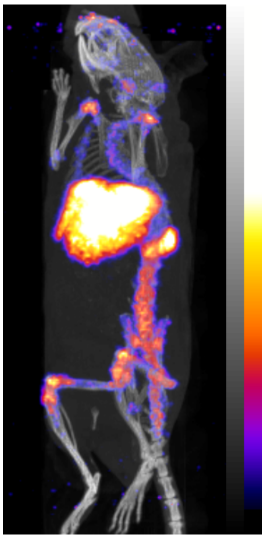

**Fig. S4** PET/CT scan of $^{89}Zr$ 14 days after i.v. inoculation of a C57Bl/KaLwRij mouse with $10^7$ eGFP-5T33 cells labelled with 5 MBq [$^{89}Zr$]-Zr(oxinate)$_4$

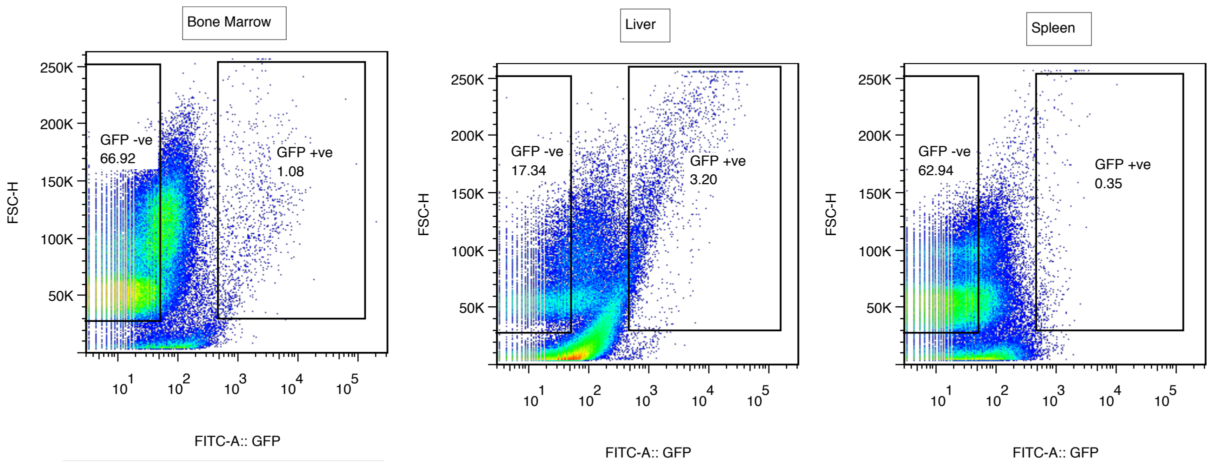


**Fig. S5** Representative FACS plots (Forward scatter (FSC) *vs.* GFP signal) of bone marrow, liver and spleen homogenates obtained from a mouse inoculated with [$^{89}$Zr]-Zr(oxinate)$_4$ labelled eGFP-5T33 cells. eGFP-positive ("GFP +ve") and eGFP-negative ("GFP –ve") populations are marked; the same gate settings were used to process every sample. Note that eGFP-positive and eGFP-negative populations are very well separated to prevent cross-contamination during FACS sorting
